# Letter from Dr. Wm. T. G. Morton of Boston

**Published:** 1847-10

**Authors:** Wm. T. G. Morton

**Affiliations:** Boston


### ARTICLE VII.

### *Letter from* Dr. Wm. T. G. Morton.

Boston, August 25, 1847.

Messrs. Editors:

During the session of the late convention of dentists at Saratoga, I found that great interest was manifested in the history of the discovery of the mode of producing insensibility to pain under surgical operations, and a strong desire expressed to have this history fully and fairly set before our profession through the columns of its own proper Journal. This desire was expressed to me personally by several of our brethren, whose rank, at the head of the profession, entitled their intimations to great weight. Yielding to this, I present a statement of some of the chief incidents relating to the discovery, from the earliest, set forth somewhat in the narrative form. After the right to the credit of the discovery became matter of controversy, affidavits were collected, the originals of which were forwarded to the Academie des Sciences at Paris. Many of them will be found in a pamphlet, published by Mr. Edward Warren, in Boston, especially in the latest editions. They are, perhaps, too long to justify their being incorporated in this article, but I transmit such as are printed to your care, to be used as your discretion shall dictate.

In the year 1844, while in practice as a dentist, I desired to improve myself in chemical and medical knowledge, and entered the family and laboratory of Dr. Charles G. Jackson of that city, and devoted my spare time to study and experiments, under his supervision. Incidentally, in the course of conversation, I spoke of killing the nerves of teeth by arsenic, and expressed some want of confidence in the practice, as it often left a permanent soreness, arising from irritation of the lining membrane. Dr. Jackson told me, humorously, to try his tooth-ache drops, and on my inquiring what they were, told me that when in the practice of medicine, some years before, he occasionally pulled teeth for his particular patients, and in one instance, finding the patient unwilling to bear the pain of an extraction, he applied ether to the tooth, to alleviate the pain, and with success. A friend of this person came to him soon after for some of his "tooth-ache drops," as he called them, but as he did not wish to engage in dentistry, he told the applicant that he had none. I made some inquiry as to the effect of ether on the system, and Dr. J. spoke of the students at Cambridge inhaling it from their handkerchiefs, and described the effect as stupefying and intoxicating. He offered to send me some of this ether, to try upon teeth, and did so, at the same time sending some to the most distinguished dentists in the city. In this conversation he made no allusion to any other effect of ether than what I have stated, and the accuracy of my statement as to this conversation is confirmed in Dr. J's own statement to Dr. Gould of Boston. I will add that the ether which Dr. J. sent me was chloric ether.

Not long after receiving this ether, I applied it to a sensitive tooth of a female patient, and found that by repeating the application for several days the irritation of the nerve was so far relieved that the tooth was filled without difficulty, and is now in good condition. By this experiment I became satisfied of the effect of ether when directly applied, in assuaging pain and deadening the nerve of a tooth. This was in the summer of 1844.

Soon after this conversation, the wife and aunt of Dr. Jackson were under my care for a series of severe dental operations, in which no little fortitude was needed. The first named lady sat in the operating chair several hours before the extraction of each tooth, showing uncommon sensitiveness, and begging to be mesmerised, or for any mode of alleviating the pain. Dr. Jackson was present at some of these sittings, and saw her distress, and often conversed upon the subject, but suggested no mode of alleviation or prevention.

My attention having been directed to the use of ether, by direct application, to sensitive teeth, I began to read upon the subject generally, and found that the inhaling of ether to produce stupefaction or intoxication was familiar among teachers and students, and that its effect was well known to be that of an antispasmodic, anodyne and narcotic. Having some of the ether left which Dr. J. had sent me, I inhaled it from my handkerchief, but did not find that it produced any marked effect, probably from there being too little of it.

In the course of the summer, being unwell, I went into the country, at Farmington in Connecticut, and while there made experiments upon the effect of ether by causing birds and other animals to inhale it. I was disappointed in the effects produced, and as my experiments were known among my friends, I was somewhat mortified, and bottled the subjects, where they remain, I presume, to this day. The books I examined upon this subject were in part from Dr. Jackson's library, and were such as are well known to men versed in chemical science. On returning to the city in the autumn, I found that my business required my constant attention, and I was not able to pursue this subject any farther at that time.

In the winter of 1844-5, Dr. Horace Wells of Hartford, Ct., formerly my partner, came to Boston and asked me to aid him in getting an opportunity to try the effect of nitrous oxide gas in producing insensibility to pain under surgical operations. I cheerfully acceded to his request, and introduced him to Dr. John C. Warren, who invited his students to remain and hear Dr. Wells' remarks at the college; and in the evening Dr. Wells administered the gas at a hall in presence of the class, and extracted a tooth, but the patient screamed for pain, the students hissed and laughed, and the whole thing was a ludicrous failure. The gas administered was the nitrous oxide, and no allusion to ether was made by Dr. Wells to me, to the physicians, or to any one else that I ever heard of. Dr. Wells left town early the next morning without seeing me, and I did not see or hear from him again until the July following, when, being in Connecticut, I called upon him at Hartford for the purpose of adjusting our former partnership accounts. I found that he had given up dentistry and had been engaged in an exhibition of birds, his health having failed him. At the office of Dr. Riggs I spoke of the gas, but Dr. Wells gave me to understand that he had abandoned the experiment, being satisfied that it could be of no practical value.

My business had now become almost exclusively mechanical dentistry or plate work, and I was required constantly to extract a great number of teeth from the same patients, who suffered extremely, being often obliged to put off my operations and sometimes to abandon them altogether. I had a motive which few other men have, daily pressing upon me, to discover some mode of destroying or greatly alleviating pain under dental operations. I knew the effect of ether, when directly applied, to render a nerve insensible. I knew the effect of it, to some extent, when inhaled. I knew of Dr. Wells' attempt to produce insensibility to pain by means of inhaling nitrous oxide gas. It was not remarkable that I gave my attention to producing this result by other means, and that I tried the gas or vapor of ether.

In the course of this summer (1846,) a student came to my office by the name of Thomas R. Spear, who, finding that I was engaged on this subject, told me that he had inhaled ether when at school, and described to me its effects. I tried an experiment upon a water spaniel, forcing his head into a jar from which he inhaled the vapor. In a few minutes he wilted down in my hands, and became apparently lifeless. He then sprang up, yelled, and leaped to some distance. This experiment incited me to go forward. I made a contract with Granville G. Hayden, a young dentist, to take the entire charge of my business, that I might give myself unreservedly to this subject. The contract was drawn on the 30th June, 1846, by Richard H. Dana, Jr., Esq., and is now in his possession. In a published letter, addressed by him to Mr. Warren, he states his recollection of the object I had in view, as explained by me to him at the time.

As soon as Dr. Hayden became established in my business, I continued my experiments. I first inhaled chloric ether with morphine, as described in the report of Dr. H. J. Bigelow, but the effect was only drowsiness, followed by lassitude and headache. Early in August Dr. Hayden, at my request, procured me a four ounce vial of sulphuric ether from Burnett, a druggist much relied upon by chemists. I tried to induce Dr. H. to inhale it, but he declined. I took half of it into the country to repeat the experiment upon my dog, but he sprang and overset the jar. Being somewhat out of patience, I resolved to take it myself, and inhaled the other half from my handkerchief. I was somewhat insensible, but not entirely so; still there was effect enough produced to encourage me. As Mr. Burnett's shop is near my rooms, and his young men are intimate with my students, I preferred not to send there again for ether lest he should learn what I was about; so I sent a student, Wm. P. Leavitt, to a druggist, in another street, who sent me a quantity of sulphuric ether, which has since been analyzed, and turns out to have been far from pure, containing large quantities of alcohol, sulphur acids, and other impurities. Not knowing this to be the case, I requested Thomas R. Spear, a student, to inhale it. As he had done so at school, he consented. The effect was at first drowsiness and torpor, followed by extreme excitement and violence. It was necessary to hold him down in his chair. Leavitt also took it, with very much the same effect. These experiments discouraged me. The results were not what I desired, nor what the experiments upon myself and on the dog led me to hope might take place.

It is important to remark here, that had the ether I used at this time been pure and highly rectified, I should unquestionably have demonstrated the great truth then, instead of on the 30th September, as I shall hereafter state.

Early in August I went into the country for health and recreation, and did not return to my labors until the middle of September. As it had occurred to me that more satisfactory results might be produced by a more appropriate inhaling apparatus, I called frequently upon Mr. Wightman, a philosophical instrument maker, for the purpose of contriving something. While examining his bags for inhaling the exhilarating gas, it struck me that by making an opening for admitting atmospheric air, to be closed by a valve, I could convert one of these into an inhaling apparatus. I asked Mr. W. if ether would dissolve India-rubber. He thought it would. I asked him if it would dissolve oiled silk. He said he did not know, but advised me to consult a chemist, and named Dr. Jackson. I got a glass tunnel from

Mr. Wightman, purchased an India-rubber bag, and returning to my office sent W. P. Leavitt to Dr. Gay, a chemist, to ask the simple question, whether ether would dissolve India-rubber? Dr. Gay was out. I became satisfied that my glass and bottle were too small for my purposes, and said to Dr. Hayden that I would step into Dr. Jackson's laboratory and get a gas-bag. Dr. H. suggested to me to ascertain from Dr. Jackson something with reference to the properties of ether. I said that I would do so, if I could without letting Dr. J. know what result I was endeavoring to produce. I went to Dr. J's laboratory to procure a gas-bag, at all events, and with the further intention of getting information from him as to the preparations and kinds of ether, if I could do so without making known to him my whole purpose. I know that some may consider this course unworthy of one engaged in a philosophical inquiry and seeking only for truth. But, on the other hand, it may justly be said that I had devoted time and money, and made great sacrifices for this object, and felt that I needed only a little specific information, which any well informed chemist could give me, and which I was willing to pay for. I had an arrangement with Dr. Jackson, ever since I studied with him, as to occasional advice I might ask of him; yet, by asking this advice, I might enable another man possessed of the requisite scientific information to avail himself of my labors and ideas, and just to come in between me and my results.

I asked Dr. J. for his gas-bag. He told me it was in his house. I got it, and returned through the laboratory with the bag in my hand. Dr. J. said, laughingly—Well, doctor, you seem to be all equipped, minus the gas. I replied, in the same vein, that there would be no need of gas, if I could make the man who took it believe he was taking gas; that imagination would do every thing, and alluded to the story of the man who died from being made to think he was bleeding to death, when there was only water trickled down his leg. Dr. J. said, that was a good story, but added, in a graver manner, that I had better not try that experiment, lest I should be set down as a greater humbug than Wells was with his nitrous oxide gas.

Finding the subject open, I said, in as careless a manner as I could assume, why cannot I use the ether gas? He said that I could do so, and spoke again of the effect of it upon the students at college, and said it would make the patient stupified and torpid, so that I could do what I pleased with him, and that he would not be able to help himself. I asked him then as to the kinds and preparations of ether, and supposing that if he had any it would be the best, I asked him to let me see his. He did so, but said that his had been standing for a long time, and recommended me to get some at Burnett's, who, he said, had it highly rectified and pure. As I went out, he told me he could supply me with something better than the gas-bag, and gave me a flask with a glass tube inserted in it.

Procuring the ether from Burnett's, I shut myself up in my room, seated in the operating chair, and commenced inhaling. I began to feel the effects, looked at my watch, and soon lost consciousness. As I recovered, I had a dreadful sensation like that of nightmare, and would have given the world for some one to come and arouse me. At one time I feared I should die in that state. Gradually, as consciousness returned, I found there was numbness and insensibility in my limbs, and bringing my fingers together, and pinching my thigh, I found there was no sensation. I then looked at my watch, and saw that I had been in that state between seven and eight minutes.

Finding every indication of a complete and successful experiment, I waited impatiently for some person upon whom I could perform an operation. At the latter part of the afternoon a man by name of Eben. H. Frost, who lives in Prince street, called and wished to have a tooth extracted, but seemed to dread the operation, and asked if he could not be mesmerised. I told him I had something better, and gave him the ether. His insensibility was perfect, and I drew from him a firmly rooted bicuspid tooth. He knew nothing that had been done, until the tooth was shown to him. Dr. Hayden and Mr. Tenny of the Journal office were present. This was on the evening of the 30th September, 1846, and I believe it is not questioned that this is the first successful experiment upon the inhaling of ether to produce insensibility under operations upon the human frame.

It may not be improper for me to make a remark, here, upon the conversation with Dr. Jackson. I do not recollect to have originated all knowledge and all ideas on this subject. I derived from Dr. Jackson, in 1844, the information that ether directly applied to a sensitive tooth would gradually produce insensibility of the nerve, and this I corroborated by experiment. At the same time he spoke of the effect of ether when inhaled, but the effects he described were only such as were detailed in the books, and well known to teachers and students. I knew of Dr. Wells' attempt to produce insensibility under surgical operations by means of nitrous oxyde gas. I had a motive for endeavoring to produce this insensibility, or, at least, the highest possible degree of alleviation of pain under dental operations, which few other persons had. It was owing to circumstances creating this strong motive, aided by the impression I had obtained, that I gave my mind to the subject, and worked out the result, demonstrating a truth, the extent and power of which have been as astonishing to me as to the rest of the world. I am ready and desirous to acknowledge my obligations to all persons from whom I have received any aid. I am indebted to Dr. Jackson, also, for information as to the best kinds and preparations of ether. But my obligations cease here. Dr. Jackson gave me no information which I could not have obtained from other well informed chemists, and from some books. He described the state which inhaling the ether would produce, as stupefaction, intoxication, torpor, and the like; effects known to scientific and medical men, but gave no intimation of the result which was actually produced, and which, alone, creates the wonder and usefulness of the discovery. His conduct at the time, and subsequently, and the affidavit of Mr. Eddy, show, conclusively, that he had not this idea or suspicion on his mind. If my experiment had produced no other result than he anticipated, it would not have been, as he evidently thought it was not, worth troubling one's-self or the world about. I knew of Dr. Wells' experiment, but this was not knowledge confined to myself, it was known to Dr. Warren, Dr. Heywood, Dr. Jackson himself, and to many physicians and students in Boston. But Dr. Wells did not allude to ether, as the surgeons he conversed with are well aware, and have stated. My position is simply this: that under the influence of a sufficient motive, and aided by ideas and facts, obtained as I have stated, of my own motion, and at my own risk, I followed up the subject and demonstrated the truth.

I waited one day after the experiment upon Mr. Frost, and called upon him to see how he was affected. Finding him perfectly well, I determiued to introduce the discovery into immediate use. My chief difficulty was, in inducing persons to try it. Mr. Frost's certificate was not enough, as he was a person unknown. I, therefore, called on Dr. Jackson, told him what I had discovered, and asked him to give me a certificate that the inhaling of this ether was harmless. He positively refused to do so, and took no part or interest in the matter. I thought then, and am now firmly persuaded, that he paid but little respect to my announcement, attributing the effect I described to imagination, or exaggeration, or both. I then called on Dr. Warren, stated to him my object, and obtained an invitation to administer it at the hospital.

During the interval between this invitation and the time appointed for the operation, I made several experiments at my office with various results. In some cases I failed, and in others a mixed and unsatisfactory result was produced, and I gave my attention to the mode of administering, and to the admission of atmospheric air. On the night before the operation at the hospital, I became very anxious, and consulted my friend Dr. Gould, a physician, who has paid much attention to chemistry, who gave me valuable assistance, suggesting an antidote in case of unfavorable effects, and recommending to me the valvular system instead of that which I had before used. From early daylight I was employed with Mr. Chamberlain, the instrument maker, until past ten o'clock, and hurrying to the hospital, reached the room just as Dr. Warren, despairing of my arrival, was about to commence the operation. The detailed account of this experiment is to be found in the communications and reports of the surgeons. It is enough to say that it was successful, and that Dr. Warren announced to the students his belief that there had been entire insensibility to pain.

The next day I administered the ether in an operation for a tumor, by Dr. Heywood, and with perfect success. I continued to administer it in my office, and early in November applied to Dr. Heywood for leave to administer it in a case of amputation, which I learned was to take place at the hospital. The surgeons informed me that they thought it their duty to decline using my preparation until informed what it was. I immediately addressed a note to the senior surgeon, informing him that the substance was sulphuric ether. The amputation took place on the 7th November. It was still undecided whether I should be admitted, and Dr. H. J. Bigelow, who interested himself a good deal in my favor, took me to the hospital, where I awaited the decision in the anteroom. Being admitted, I gave the ether with perfect success. This was the first case of a capital operation. Dr. Jackson knew nothing of this operation, being out of the city at the time, and was in no way connected with the business to the knowledge of the surgeons. On the 21st November, I administered the ether again, in an operation for a tumor, at the Broomfield House, in the presence of a number of medical gentlemen, among whom was Dr. Jackson. This was the first time Dr. J. had seen the ether administered, and no one had then administered it but myself. In this instance he was merely a spectator among others. On the 2nd January, 1847, Dr. Jackson called at the hospital, and recommended oxygen gas as a remedy for asphyxia, which he heard was produced by the ether. But the surgeons told him that they had long been satisfied that asphyxia was not produced. With the exception of a single intimation mentioned in Dr. Warren's communication, this was the first time the surgeons at the hospital knew or saw Dr. Jackson in connection with this discovery. They had no reason to suppose that he had any connection with it, and his conduct and conversation with various persons in the interval between the 30th September, and the full and acknowledged establishment of the discovery, shows, abundantly, that he did not then wish to be identified with it. I have made no regular effort to procure this evidence, and as much of it is conversation, gentlemen are unwilling to volunteer testimony upon it. And I will remark here, once for all, that until the return from Paris of Dr. Jackson's communication to the French Academy, in which he took to himself the entire credit of the discovery, not mentioning my name, that I had any suspicion that my right as discoverer was disputed, or that I had a rival in the field. I, therefore, took no pains to secure evidence in the early stages of the discovery, and much that I now have has come in almost accidentally, piecemeal, and volunteered. I have not yet made a thorough collection of evidence, but am authorised to say that there is some at my disposal of a conclusive character, which I am not at liberty to use, unless it shall be absolutely necessary. Indeed, so entirely was Dr. Jackson disconnected with the discovery, that had it failed, none of the ridicule would have been attached to him, and had a life been lost, or any serious injury been done, none of the odium would have fallen upon him, nor could he have been made in any way responsible. It was not until after the formidable opposition organised against it had become powerless, and success was certain, that Dr. Jackson began to bring forward his claims as a discoverer.

On the 17th October, I addressed a letter to my former partner, Dr. Wells, informing him of what I had done, and inviting him to come to Boston and assist me in bringing the discovery into use. He replied by a letter of October 20th, speaking encouragingly of my discovery, and saying that if the gas will produce the effect I named, it will be a fortune to me, and intimating no claim to a prior discovery. His letter is printed and transmitted with this article. In a few days he came to Boston, saw a number of operations in my office, in the early stage of the discovery, was told freely, and could discern by the smell, that it was ether I used, yet gave no intimation of having used it himself. On the contrary, he seemed disappointed with the results, expressed a fear of the consequences, and said that I

should kill some one, or, at all events, injure myself very much in my business. He left Boston, and soon went to Europe. While there the fame of the discovery reached him, and then, for the first time, he put in a claim for the merit of a discovery, professing not to know that I made pretensions to the same honor. I have the authority of several surgeons for saying, that he did not allude to ether when in Boston, in 1845, and of Dr. Geo. Hayward for saying that Dr. Wells, since his return, admitted to him that he had never made any experiment with ether.

I feel bound to make an explanation on the subject of the patent which I obtained. My intention had been chiefly to discover something which I could use to advantage in dental operations. The other uses to which it could be put were collateral to my purpose, and I had no desire to interfere with them. But I was advised that if I did not procure a patent, my discovery and the instruments by which I administered the ether would be used by other dentists, while I, who had devoted my time, money and labor to it, and had run all the risks and encountered all the opposition, odium and ridicule, would receive no greater reward than they. I did not at the time fully realize the manifold uses and extraordinary powers which have since been developed. I was also advised that this would be a dangerous instrument for evil, and that public policy would be subserved by confining the use to responsible persons. I immediately presented the right to all charitable institutions in the United States, together with apparatus to many of them, and made it known that I would give every facility for its use for the smallest compensation in surgical cases, and would sell rights to dentists for a consideration which all would admit to be reasonable. My object was to secure to myself a reasonable return for the discovery and for all that I had undergone and risked in its prosecution. I can sincerely say that I now regret having taken this step, but a man's motives should not be judged by the result; he must be judged according to the circumstances under which he acted. As I have now abandoned all intention of adhering to the patent, I wish also to have the result of my efforts understood. So far from having gained, I have been a pecuniary loser by this discovery. It took me from my business, which I was obliged to confide to other hands, involved me in outlays bringing no return, and in personal controversies, and now the discovery is freely used by those very persons whose opposition caused me for a time so much loss and inconvenience.

If the question is asked, how Dr. Jackson came to be associated with me in the patent, I refer the reader to the letter of R. H. Eddy, Esq., the patent agent, in answer to one addressed to him by the surgeons of the hospital, which developes the history of that transaction in a manner which I cannot doubt will be satisfactory. From that letter it will appear that Mr. Eddy had but a slight acquaintance with me, and was an intimate friend of Dr. Jackson, having perfect confidence in him and a strong interest in his success. When Dr. Jackson found I was to get out a patent and heard that I was likely to make a great deal of money from it, he made me a professional charge for advice as to the preparations of ether, &c., which he had given me. Mr. Eddy then suggested to me that instead of paying Dr. Jackson a fee, I should allow him a small interest in the patent. He gave as a reason for this that I should thus have the aid of his name, which would be a great help to me in the opposition then directed against me, and give a scientific character to the discovery, and chiefly because Dr. Jackson would then have a motive for giving his attention to the subject, would suggest improvements in the application and preparation of the ether, and keep in advance of the improvements which might be made by others. I felt the need of all aid I could get in my then situation, and the want of that scientific skill which intense competition would render necessary to the value of the patent, and agreed to substitute a share in the profits of the patent for a fixed fee. Mr. Eddy also had another motive for securing Dr. Jackson. He knew that the patent would inevitably be contested, and that Dr. Jackson would as certainly be a witness at any trial that might take place. At that time he knew but little of the history of the discovery, and from his general confidence in Dr. Jackson, was inclined to magnify his share of the business. He therefore inferred that Dr. Jackson's testimony in court, and statements out of court, would be of such a kind as might give a handle to the opponents of the patent to deny my exclusive right, and at least to embarrass the cause. He therefore thought it would be good policy for me to admit him into the right. Mr. Eddy states that I protested against admitting Dr. Jackson to have been a joint discoverer, but that my objections were overborne by his arguments and by his opinion, which he then entertained, that Dr. Jackson, by reason of the advice he gave me, was entitled to a share in the merit of discovery. I was entirely ignorant of the principles of patent law, and knew that Mr. Eddy had made that his chief study and his sole business. I therefore reluctantly assented to the arrangement. Mr. Eddy now says, distinctly, that if he had then known the history of the transactions between Dr. Jackson and myself, he not only would not have advised, but would not have permitted Dr. Jackson's name to be associated with the discovery. I respectfully request the attention of every reader to this letter, as well as to that of Mr. Caleb Eddy upon this particular point. They are to be found in the later editions of Mr. Warren's pamphlet, and are transmitted with this communication. W. T. G. MORTON.

"*Hartford, (Conn.,) Oct.* 20, 1846.

Dr. Morton :

Dear Sir—Your letter, dated yesterday, is just received, and I hasten to answer it, for fear you will adopt a method, in disposing of your rights, which will defeat your object. Before you make any arrangements whatever, I wish to see you. I think I will be in Boston the first of next week—probably Monday night. If the operation of administering the gas is not attended with too much trouble, and will produce the effect you state, it will, undoubtedly, be a fortune to you, provided it is rightly managed. Yours, in haste,

H. WELLS."

***Correspondence between the Surgeons of the Massachusetts General Hospital, and Caleb Eddy, Esq., and Mr. R. H. Eddy.***

Since writing the foregoing, the wish therein expressed has been gratified; and, by the kindness of Drs. Hayward, Townsend, Parkman, and Bigelow, surgeons of the Massachusetts General Hospital, I am permitted to lay before the public the following correspondence between them, and Caleb Eddy, Esq., and Mr. R. H. Eddy. As these communications will speak for themselves, I need not add any comments, but will give them at once, merely expressing the pleasure it affords me, thus to be able to adduce a kind of testimony which cannot fail, as it seems to me, to carry conviction to the minds of the most incredulous. This correspondence affords me the means of corroborating many of my foregoing statements.

The subjoined is the letter from three of the surgeons, Dr. Bigelow having addressed a separate note to the same effect, which, therefore, I will omit:—

*Boston, May* 18, 1847.

Caleb Eddy and R. H. Eddy, Esqs.

Gentlemen,—The undersigned, having been informed that you are in possession of important information relative to the discovery of the new property of sulphuric ether, and of its subsequent history, are desirous that you should, at your earliest leisure, furnish them with such an account of the matter as will elucidate so important a subject.

They will thank you to state how the names of Dr. Charles T. Jackson and Dr. W. T. G. Morton became associated in the letters patent; what share each had, in your opinion, in making the discovery; and any other facts you may choose to communicate tending to the same end.

GEO. HAYWARD,<br>

S. D. TOWNSEND,<br>

SAMUEL PARKMAN,<br>

*Surgeons of the Massachusetts General Hospital.*

*Boston, May* 24, 1847

To Drs. George Hayward, S. D. Townsend, Samuel Parkman, Henry J. Bigelow, } Surgeons of the Mass. General Hospital:

Gentlemen,—Your favors of May 18th and 20th, addressed to myself and Mr. R. H. Eddy, have been received. As I presume any reply I may make will be made public, I would take the occasion to remark, that, were it not that there now seems no possibility of the controversy existing between Drs. W. T. G. Morton and C. T. Jackson being settled by mutual arbitrament, owing to the refusal of the latter to submit the same to a reference, I should feel an indisposition to make any relation of what came under my notice relative to the discovery in question. I have no wish to rob Dr. Jackson of any honor to which he may be properly entitled, and am governed by no interest further than a desire that he to whom the world is really indebted for making the discovery may receive that reward to which he is justly entitled.

On the evening of Friday, October 23, 1846, Dr. Charles T. Jackson visited my house. During the evening, I requested him to relate to me the particulars of the new discovery for prevention of pain in surgical operations. He stated to me, that Dr. W. T. G. Morton called on him near the latter part of last month to obtain the loan of a gas-bag, which he said it was his intention to use for the purpose of administering atmospheric air, or something else, to a patient to quiet her fears in order that he might extract one of her teeth: that he informed Dr. Morton that his gas-bags were in the attic story of his house, and it would be attended with some trouble to procure them: that Dr. Morton stated that he was desirous of operating on the imagination of the person in some such way as was said to have been practised on a criminal condemned to death, viz.—by suffering warm water to trickle upon and from some wounded or lanced part of the body, while the eyes of the person were bandaged. Dr. Jackson stated, that he told Dr. Morton that such an experiment would prove a failure, and he would be ridiculed for making it; that he had better let her breathe some ether, (if he could induce her to inhale it,) which would put her to sleep, and then he could pull her tooth, and she could not help herself, or could not prevent him by any resistance: that Dr. Morton inquired of him as to the danger and mode of using it. He replied to him, that he might saturate a sponge or cloth with it, and apply it to her mouth or nose. After Dr. Jackson had related the above, I said to him, "Dr. Jackson, did you know at such time, that, after a person had inhaled ether, and was asleep, his flesh could be cut with a knife without his experiencing any pain?" He replied, "No! nor Morton either: he is a reckless man for using it as he has; the chance is, he will kill somebody yet." This is all, or nearly all, of any importance, that I now recollect in relation to the discovery, previous to the application for the patent in which the names of Drs. Morton and Jackson were associated.

With respect, your obedient servant,

CALEB EDDY.

*Commonwealth of Massachusetts,*
*County of Suffolk, City of Boston.*

Be it known, that, on this twenty-ninth day of May, Anno

Domini eighteen hundred and forty-seven, before me, the undersigned, a notary public, within and for said county and city, duly commissioned and sworn, came Caleb Eddy, and made solemn oath that the foregoing statement, by him subscribed, is true. The said Caleb Eddy is well known to me as being one of the most respectable and trustworthy citizens of said Boston.

In witness whereof, I have hereunto set my hand and seal notarial.

JOHN P. BIGELOW, *Notary Public.*

*Boston, May* 22, 1847.

To Drs. Geo. Hayward,<br>

S. D. Townsend,<br>

Samuel Parkman,<br>

H. J. Bigelow,<br>

Surgeons of Mass. General Hospital:

Gentlemen,—I have received your communications of the 18th and 20th instant, in which you state that you have understood me to be "in possession of important information relative to the discovery of the new property of sulphuric ether, and of its subsequent history," and are desirous that I should "furnish such a statement of the matter as will elucidate so important a subject;"—also, "to state how the names of Drs. C. T. Jackson and W. T. G. Morton became associated in the letters patent, and what share each had, in my opinion, in making the discovery." "Also, any other facts I may choose to communicate tending to the same end."

The friendly relations which, for many years, have existed between myself and Dr. C. T. Jackson, have heretofore caused me to refrain from making known many facts in my possession in relation to the late discovery of the new effect of sulphuric ether. The difficulties, between him and Dr. W. T. G. Morton, I hoped to see settled by an impartial reference—one, where the evidence, produced by both parties, could be subjected to a rigid examination, in order that truth might be elicited, and strict justice rendered to whichever of those gentlemen such a tribunal should accord the chief merit of making the discovery. I have earnestly recommended Dr. Morton, whenever an opportunity has presented, to induce Dr. Jackson to submit the matter of the discovery to such a reference. Accordingly, it was a cause of much gratification to me to learn that a proposition of Dr. Morton to do so, had received the favorable consideration of Dr. Jackson. I find, however, my anticipations have not been realized. Dr. Jackson, after having consented to refer the case, and after delaying, a long time, to agree on a suitable umpire, has, as I learn, utterly refused to submit his claims to a just arbitration. Under such circumstances, I feel it a duty to make known to you a few facts. My business engagements prevent me from stating a particular history of much that has come under my observation in relation to this matter. I shall, therefore, endeavor to confine myself to a simple statement of what I was witness to, from the time I first heard of the discovery until a patent was applied for in this country.

Within a few days of Sept. 30, 1846, I think the 1st of October, Dr. W. T. G. Morton called on me at my office, stated to me that he had made an important discovery, by which he could extract teeth without pain, and desired to learn from me whether it could be secured by a patent. After replying to him that he must state the nature of it, before I could render him any definite opinion, he informed me, that he used sulphuric ether, by administering it by inhalation in a state of vapor. He mentioned, that he had extracted a tooth without the patient being sensible of the operation, and that, on awakening from the sleep into which he had been thrown, he was much surprised to find his tooth drawn and lying on the floor.

I stated to Dr. M. that, as to the patentability of the discovery, I had some doubts; but that I would consult the law, and the various legal decisions on the subject of patents, and advise him of the result. After this, I saw Dr. Morton not more than once, I think, if once, until Wednesday, the 21st day of October. In the mean time, I had read several articles in the newspapers relative to the experiments performed at the Massachusetts General Hospital, and had understood, from Dr. Charles T. Jackson, that he had had some connection with Dr. Morton in making the discovery. My reflections on the subject led me to the belief, that a patent could be obtained in this country, and on the 21st day of October, Dr. Morton having called at my office, I so informed him. I stated to him that, from what I had learned from Dr. Jackson, I considered the discovery to be a joint one, and that the patent, if applied for, must be conjointly by him and Dr. Jackson. In rendering such advice, I was fully impressed with the belief, from the statements of Dr. Jackson, that he, Dr. J., had suggested to Dr. Morton the propriety of experimenting with ether,—that Dr. Morton, without the presence or further assistance of Dr. Jackson had practically demonstrated the effect of ether to annul pain. Upon this I reasoned, that, had Dr. Morton kept the discovery secret, neither Dr. Jackson nor the world would have known of the result; or, in other words, had Dr. Morton not performed the experiment that he did, the discovery made could not have taken place;—also, that had not Dr. Jackson given Dr. Morton the *idea* of using ether, neither Dr. Morton nor the world would have known of the discovery. It seemed to me to be a clear case of joint invention or discovery. Dr. Jackson had admitted to me that he had never performed a surgical operation of any kind on a patient, under the influence of inhaled ether.

In reply to my remarks to Dr. Morton, he stated that he did not know by what right Dr. Jackson should have any interest in the patent, as he (Dr. M.) had an understanding with Dr. Jackson to fully remunerate him for any advice he might have rendered him. In order to satisfy myself more fully as to the position of Dr. Jackson in this discovery, and the understanding between him and Dr. Morton, I called at the office of Dr. Jackson the next morning. I cannot recollect the precise conversation which ensued at this interview, but the substance of it was, that Dr. Jackson informed me that, by the laws of the Massachusetts Medical Society, he would be prevented trom joining with Dr. Morton in taking out a patent, as he would be expelled from the association if he did so. He further stated, that he intended "to make a professional charge of $500" to Dr. Morton, for the advice he had given him, and that Dr. Morton had acceded to this; that he did not wish his name connected with Dr. Morton's in any manner; that Dr. Morton might take out a patent if he desired to, or do what he pleased with it. I made inquiries as to the assistance rendered Dr. Morton, and asked Dr. Jackson if he had ever tried any experiments to practically demonstrate the fact that the inhalation of ether would prevent pain during a surgical operation. He informed me that he had not. I am fully persuaded that Dr. Jackson, at this time, thought the whole matter of little value or importance. The conversation I had with him led me to this belief. He supposed Dr. Morton might realize something from it in his business of dentistry, and was willing that he should do what he pleased with it, so long as he did not couple his (Dr. J's) name with it. I afterwards inquired of Dr. Morton whether he had agreed to give Dr. Jackson $500 for the assistance rendered, as well as for all the Doctor's interest whatever in the discovery. He said that he had, and that he had agreed to pay him at the rate of *ten per cent.* on the sale of licenses, until the $500 was paid.

On Friday evening, Oct. 23d, on my return to my residence after a visit to the theatre, I found Dr. Jackson in conversation with my father, Caleb Eddy, Esq., and waiting to see me. At this interview, I urged Dr. Jackson to waive his objections to associating with Dr. Morton, as I was confident that he was mistaken in his views of what would be the action of the Medical Association; that Dr. Morton could not properly take out a patent without him; and that, by joining in the patent, he would, of a certainty, be obtaining *credit as a discoverer;* whereas, should he not do so, he might lose all credit, as in the case of the Magnetic Telegraph, which I had understood from Dr. Jackson he had suggested to Professor Morse.

The next day, or within a few days after, I called on Dr. Augustus A. Gould, to learn from him the nature of the rules of the Medical Society. Dr. Gould I knew to be a personal friend and a well-wisher of Dr. Jackson. He exhibited to me a copy of the by-laws, in which I found they only provided, so far as I could see, that no member should deal in *secret remedies.* I perceived at once from them, that no objection could arise to Dr. Jackson's patenting any invention he might make, as it would cease to become *secret* the moment it might be *patented.* I understood Dr. Gould to coincide with me in my views. After preparing the specification, I submitted it to Dr. Jackson, who fully approved it. I next had it copied in a manner suitable to be signed and sworn to by the parties.

I recommended to Dr. Morton to allow me to insert, in the written agreement to be made between him and Dr. Jackson, ten per cent. on all sales of licenses, instead of ten per cent. until the amount to be paid would reach $500; advised him to be liberal towards Dr. Jackson, both in giving him credit, and a chance of profit. In this, I was governed by a sincere desire to benefit Dr. Jackson, while, at the same time, I supposed I was doing my duty to Dr. Morton, as I believed it would be for his interest to do so. I thought the chemical science of Dr. Jackson would be brought to improve the article used, or to produce a better quality of ether than could be found in the market; that his association with Dr. Morton would give immediate character to the discovery, and his future advice might be of great service to Dr. Morton.

My views seemed to strike Dr. Morton very favorably, and he acquiesced in them.

Here I would remark that he (Dr. M.) had never *informed* me of any experiments with ether, which I have since understood he made previous to his obtaining advice in relation to it from Dr. Jackson. This I can readily account for, as I saw very little of him, from the 21st to the 27th of October, the latter being the day on which the papers for the application for the patent were executed by the parties.

Dr. M. was so much engaged in his discovery and business of dentistry, that I found it exceedingly difficult, if not impossible, to obtain an audience with him. His office was constantly thronged with persons in waiting to consult him on professional and other business. Had Dr. Morton, during this time, stated to me what I have since read in the affidavits of Dr. G. G. Hayden, Messrs. W. P. Leavitt, T. R. Spear, Jr. and F. Whitman, I am confident I never should have advised him to associate Dr. Jackson in the discovery or patent, as I should have concluded that his friendly intimacy with Dr. Jackson had led him to visit him, as the readiest manner of obtaining certain chemical information respecting ether and its properties, which might be found in various scientific or medical works not conveniently accessible to him.

I should have considered that the idea of using ether was an original one with Dr. Morton; that he had, by a practical application of it, made the discovery that it would annul pain under the operation of a surgical instrument; had been the first to publish this to the world, and under peculiar circumstances, in which he had developed much of that remarkable energy of character we often find to belong to most great inventors, who are generally obliged to stem a powerful current of difficulties and risks, in order to impress on the community the importance of their discoveries. With such views, I do not hesitate to affirm that I should have accorded the discovery to him.

On Tuesday morning, the 27th of October, Drs. Morton and Jackson executed the papers for the American patent. While Dr. Jackson was passing from his office to my own, I informed him that I had seen Dr. Gould, and he had shown me the laws of the Medical Association; that Dr. G's opinion and mine coincided as to what was meant in them by the prohibition of secret remedies; that such could not be *patented* ones, as *they* (the latter) could not be secret. He replied, "Well if Dr. Gould thinks so, that settles the matter with me. I have no objections to signing the papers with Dr. Morton." I think I give nearly, if not exactly, the words made use of by him.

I would here remark, that I had found Dr. Jackson tinctured with old and exploded prejudices against patents, and I labored to remove them. So successful was I, that he subsequently informed me, that, after a consultation with a distinguished chemist at the south, he had resolved to secure every invention he might hereafter make; and, in accordance with such views, he sent me the specification of an alleged improvement in preparing a certain article for dentistry purposes, with the view of filing a caveat and taking out a patent on the same. His disinclination to associate with Dr. Morton, in a patent, arose from no disposition, ever evinced to me, to give the public a *gratuitous* use of the discovery. The most important objection to his taking out a patent arose from what he supposed would be the action of the Massachusetts Medical Association.

In conclusion, I would remark that I have endeavored to state a few facts relative to the early discovery of the effect of sulphuric ether in surgical operations. In doing so, I am influenced by no other motives than to render justice to whom it may be due. It is a matter of indifference to me to whom the world may ultimately accord the merit of being its benefactor for having given to it the great discovery in question. Dr. Jackson has been my personal friend for many years. With Dr. Morton, I have had, comparatively, but little acquaintance, never having seen or known him previous to my introduction to him while he resided in the family of Dr. Jackson. My sympathies would naturally tend towards Dr. Jackson; but personal friendship, private character, or scientific attainments, are matters which, it seems to me, ought not to prejudice me or any one else in favor of or against either of the claimants, when judging of the merits of their respective claims.

Yours respectfully,

R. H. EDDY.

*Commonwealth of Massachusetts,*

*County of Suffolk, City of Boston, ss., June* 18, 1847.

Then personally appeared the above-named R. H. Eddy, and being duly sworn, did declare that his statements, contained in the foregoing letter, by him subscribed, are true, according to the best of his recollection, knowledge, and belief.

Before me,

JOHN P. BIGELOW, *Justice of the Peace.*

